# Major reduction of malaria morbidity with combined vitamin A and zinc supplementation in young children in Burkina Faso: a randomized double blind trial

**DOI:** 10.1186/1475-2891-7-7

**Published:** 2008-01-31

**Authors:** Augustin N Zeba, Hermann Sorgho, Noël Rouamba, Issiaka Zongo, Jeremie Rouamba, Robert T Guiguemdé, Davidson H Hamer, Najat Mokhtar, Jean-Bosco Ouedraogo

**Affiliations:** 1 Institut de recherche en sciences de la santé (IRSS), Bobo Dioulasso, Burkina Faso; 2 Centre Muraz, Bobo-Dioulasso, Burkina Faso; 3 Center for international health and development, Boston university school of public health, Boston, USA; 4 International atomic energy agency, Vienna, Austria

## Abstract

**Background:**

Vitamin A and zinc are crucial for normal immune function, and may play a synergistic role for reducing the risk of infection including malaria caused by *Plasmodium falciparum*.

**Methods:**

A randomized, double-blind, placebo-controlled trial of a single dose of 200 000 IU of vitamin A with daily zinc supplementation was done in children of Sourkoudougou village, Burkina Faso. Children aged from 6 to 72 months were randomized to receive a single dose of 200 000 IU of vitamin A plus 10 mg elemental zinc, six days a week (n = 74) or placebo (n = 74) for a period of six months. Cross-sectional surveys were conducted at the beginning and the end of the study, and children were evaluated daily for fever. Microscopic examination of blood smear was done in the case of fever (temperature ≥37.5°C) for malaria parasite detection.

**Results:**

At the end of the study we observed a significant decrease in the prevalence malaria in the supplemented group (34%) compared to the placebo group (3.5%) (p < 0.001). Malaria episodes were lower in the supplemented group (p = 0.029), with a 30.2% reduction of malaria cases (p = 0.025). Time to first malaria episode was longer in the supplemented group (p = 0.015). The supplemented group also had 22% fewer fever episodes than the placebo group (p = 0.030).

**Conclusion:**

These results suggest that combined vitamin A plus zinc supplementation reduces the risk of fever and clinical malaria episodes among children, and thus may play a key role in malaria control strategies for children in Africa.

## Background

Malaria caused by *Plasmodium falciparum *remains a major cause of morbidity and mortality among African children [1]. Between 300 to 500 millions new cases of malaria, primarily due to *P. falciparum*, are annually observed in the world, with 90% in sub-saharan Africa, and these account for an estimated one million children deaths [2]. Furthermore, resistance to drugs and insecticides used to fight this disease has hampered malaria control efforts [3]. Children and adults living in malaria-endemic areas often have a high prevalence of malnutrition and deficiencies of micronutrients such as vitamin A and zinc; this situation creates a complexity of interactions with serious health consequences [4,5].

Vitamin A is essential for normal immune function [6], suggesting that it could play a role in protection against malaria. Zinc is essential for many biological functions such as protein synthesis, growth and immunity [7]. Vitamin A metabolism requires normal zinc status, explaining the frequent association of their deficiency [8]. Beneficial protective effects of vitamin A or zinc on malaria-related morbidity have been demonstrated in Papua New Guinea, Peru and Zanzibari a [9-12]. Two randomized, placebo-controlled trials conducted in Ghana (both reported in a single publication) did not find an overall significant effect of vitamin A on malaria parasitemia rates or parasite densities although the studies showed a reduction of 23% and 32% of probable malaria illness in supplemented children [13]. However, the number of children with probable malaria was so small that this study lacked adequate power to demonstrate an effect of vitamin A on slide-confirmed malaria morbidity. In contrast to these studies that have suggested potential protective effects of vitamin A or zinc supplementation, a randomized, placebo-controlled trial in Burkina Faso of zinc supplementation failed to show a protective impact on malaria episodes [13].

Given the evidence of a positive effect of these two micronutrients when used individually on malaria [13,14], we hypothesized that, due to the potentially synergistic effect of vitamin A and zinc on immune function, dual supplementation with these two micronutrients would decrease morbidity due to *P. falciparum *malaria. We therefore performed a randomized, double-blind, placebo-controlled trial to assess the efficacy of combined of vitamin A and zinc supplementation on morbidity due to *P. falciparum *among young children in a malaria-endemic area of Burkina Faso.

## Methods

### Study site

The study was done in Sourkoudougou village in Dandé health district, which is located 25 km from Bobo Dioulasso city, Burkina Faso. Malaria is holoendemic, with transmission peaking during the rainy season (May-October) and entomological inoculation rate of 697 infected bites per person per year [15]. The village has a government health centre where the children were examined.

### Study design

#### Participants

Children aged from 6 to 72 months were recruited. Eligibility criteria were: 1) age between 6 and 72 months; 2) plan to reside in the village for at least the next 6 months; 3) middle upper arm circumference (MUAC) greater than 12.5 cm; 4) no ocular signs of vitamin A deficiency or history of night blindness; 5) no apparent chronic or debilitating condition; and 6) parental consent for the child's participation. At enrolment each child underwent a medical examination to detect malaria infection or other illness and all children received anti-malarial treatment with sulfadoxine-pyrimethamine (SP) [16] in order to clear any *Plasmodium *infection 7 days before the initiation of supplementation. History of fever, cough, diarrhea, and stomach ache, night blindness, and the use of mosquito net on the previous night were recorded. Axillary temperature, spleen size, MUAC, height (or length) and weight were also recorded. Hemoglobin concentrations were measured and thick and thin blood slides were prepared. The study was reviewed and approved by the institutional review board of Centre Muraz. An oral consent was obtained from parents or guardian of children before enrolment.

#### Randomization and micronutrients distribution and follow up

After enrolment, children were individually allocated vitamin A plus zinc or placebo in two blocks by computer-generated randomly permutated codes. The supplements and placebos were packaged and provided by U-Pharma of IRSS, Ouagadougou, in accordance with the random numbers generated. All the capsules were indistinguishable in term of color odor or appearance. Capsules were encoded for the two groups and these codes were kept off site in a secure place that could not be accessed by the study team, clinical staff, field workers, and parents. In the supplemented group each child received a single capsule of vitamin A contained 200 000 IU in 200 μl groundnut oil and a capsule of zinc containing 10 mg of elemental zinc, 6 days a week during a 6-month period. In the placebo group each child received a single capsule containing groundnut oil alone (vitamin A placebo) and a daily placebo that consisted of a zinc-free capsule with maize powder (zinc placebo), 6 days a week during 6 months.

#### Cross sectional survey and follow up

Two surveys were done – before and at the end of the study. During these surveys the following data were collected:

• Clinical & anthropometric data: history of fever, immunization data, physical examination, height and weight

• Blood samples for hematology analysis and malaria parasite detection

During the follow up period, each child was visited daily by a supplementation agent for zinc administration, and for recording axillary temperature and any sign of illness. Each child with fever (≥37.5°C) or history of fever had a blood smear performed for malaria diagnosis and the parents were invited to bring their child to the health centre for clinical examination and treatment by the nurse. An episode of *P. falciparum *malaria was defined as temperature ≥37.5° C, accompanied by the presence of asexual forms of the parasite on blood smear and no other obvious cause for the illness. Malaria episodes were treated with SP. Clinical examination, thick and thin blood smears were repeated 3 and 7 days after the first positive smear.

### Laboratory procedures

Thick and thin Giemsa-stained blood films were reviewed for the presence of *Plasmodium *species. The parasite count per μl was done by counting 200 white blood cells and the number expressed on the basis of 8000 WBC per μl [17]. Each film was examined by microscopy by a technician in the field, and re-examined by two experienced laboratory technicians and checked by a third investigator in cases of 1,5 discrepancy. Hemoglobin concentrations were determined by complete blood count with an automated counter (Coulter counter T540, Florida, USA).

### Sample size

The primary outcome was the number of clinical episodes of *P. falciparum *malaria. Assuming a prevalence rate of 50%[18] of malaria episodes in the placebo group with 80% power and α = 0.05, we hypothesized that combined vitamin A and zinc supplementation would reduce the prevalence rate by 50%. In this regard we calculated the sample size (n = 75) for each group with a consideration that 10% of participants would be lost to follow up. A total of 150 children were randomly selected at the start of the study.

### Data processing and analysis

The number of *P. falciparum *malaria episodes was the primary outcome. Malaria was defined as axillary temperature ≥37.5°C with any level of parasitemia. Secondary outcomes included the prevalence of other causes of morbidity, and all cause mortality. The prevalence of diarrhea, fever, and cough was calculated by the number of child days of the respective disease divided by the total number of days of observation. The geometric mean parasite density, hemoglobin concentration, and anthropometric measurements were expressed as mean values.

The relative risk (RR) of *P. falciparum *malaria in children supplemented with vitamin A and zinc was calculated as the ratio of incidence densities between the supplemented group and placebo group. To exclude recrudescent malaria episodes, the individual observation time was defined as the interval from the first to last day of observation minus 14 days for each defined episode. Student t tests and chi square tests were used to compare continuous and categorical variables, respectively. Wilcoxon test was used for non-normal data. The Cox proportional hazards analysis (Kaplan-Meier curve) was used to assess the effect of combined vitamin A and zinc supplementation on the time of appearance of first malaria episode. The analyses were done with SPSS 11.0. Stata 8.0 was used for the calculation of geometric mean parasite density. Epi Info™, version 3.3.2, was used to analyze anthropometric data. A p-value < 0.05 was considered as statistically significant.

## Results

**Figure 1:**
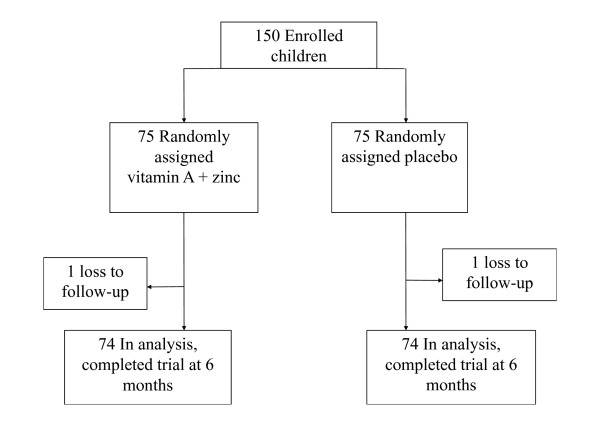
Trial profile.

One hundred fifty children were randomized (figure 1); 2 children were unavailable for analysis because they migrated out of the study area (1 in each group). The baseline characteristics of study children are given in Table 1. The supplemented (n = 74) and placebo groups (n = 74) were similar in terms of age, health centre attendance, bed net use, and anthropometric indexes. The prevalence of *P. falciparum *was higher in the vitamin A plus zinc group relative to the placebo group at baseline [76.0% vs. 61.0 % (p = 0.05)]. There was no difference in mean parasite density, spleen enlargement, or mean hemoglobin concentrations at baseline although the prevalence of severe anemia was higher in the vitamin A and zinc supplemented group.

**Table 1:** Baseline and end of study characteristics of the study population

	Baseline			End of study		
	
	Placebo (n = 74)	Supplemented (n = 74)	p-value	Placebo (n = 74)	Supplemented (n = 74)	p-value
**Age (months)**	35.2 ± 20	37.9 ± 20	0.72	-	-	-
**Sex**						-
**Female**	45.9%	48.6%	0.74	-	-	-
**Antecedent**						
Reported children with fever episode on the previous year^‡^	78.30 (69–87.6)	89.20 (82.1–96.3)	0.041	-	-	-
Reported children with mosquito-net use on the previous night	52.7 (41.4–64.0)	56.7 (45.4–68.0)	0.62	-	-	-
**Anthropometry**						
Weight-for-height Z- score <-2.0	-0.73 ± 2.8	-0.82 ± 1.4	0.58	-1.10 ± 2	-1.03 ± 1.3	0.80
Height-for-age Z- score <-2.0	-1.60 ± 2.3	-1.50 ± 2.0	0.77	-1.5 ± 2.4	-1.49 ± 1.7	0.97
Weight-for-height Z- score <-2.0 (%)	20.3 (11.2–29.4)	13.5 (5.7–21.3)	0.20	27.0 (17.0–37.0)	23.0 (13.5–32.5)	0.56
Height-for-age Z- score <-2.0 (%)	47.3 (36.0–58.6)	40.5 (29.3–51.7)	0.40	46.0 (34.7–57.3)	35.0 (24.2–45.8)	0.20
**Hematology**						
Mean (SD) hemoglobin (g/dl)	9.4 ± 1.6	9.31 ± 2.3	0.75	10.21 ± 1.82	10.09 ± 1.97	0.70
Anemia ≤ 7.0 g/dl^‡ ^(%)	5.4 (0.4–10.5)	18.0 (9.3–26.7)	0.020	5.4 (0.4–10.5)	5.4 (0.4–10.5)	0.71
**Malariometric indices**						
Positive *P. falciparum*	61.0 (51–72)	76.0 (66.-85)	0.052	57.5% (46.2–68.8)	42.0% (32.8–55.2)	0.048
Geometric mean density *P. falciparum *positive asexual forms/μl	1444 (923–2259)	1589 (1026–2459)	0.38	1945 (1155–3275)	1011 (647–1579)	0.85
Enlarged spleen	61.0% (51–72)	58.0% (47–69)	0.73	57.0% (46–68)	53.0% (42–64)	0.62

Mean ± SD or % (CI 95%)

^‡ ^p < 0.05

At the end of the study, the prevalence of malaria was significantly lower in the supplemented group (34% vs. 3.5%), respectively (p < 0.001). The mean parasite density was higher in the placebo group compared to the supplemented group at the end of the study (p = 0.048). Considering the variation of the parasite density within each group, we observed a significant decrease in the supplemented group from 1589 [1026–2459] to 1011 [647–1579] (p < 0.001) and a significant increase in the placebo group from 1444 [923–2259] to 1945 [1155–3275] (p = 0.023). At the end of the study, there was no difference in the proportion of children with splenomegaly between the two groups. Regarding nutritional parameters we observed that wasting and stunting continued to be severe in both groups (Table 1) and there was no change in the supplemented or placebo groups, respectively, at the end of the study. Mean hemoglobin rates were also similar in the two groups, and did not change significantly after six months. The proportion of children with anemia decreased significantly in the supplemented group from 18.0% to 5.4% (p = 0.02) whereas there was no change in the placebo group.

The weekly surveillance data showed that the mean number of episodes of fever episodes per child was significantly higher in the placebo group (Table 2). Children in the placebo group had a greater risk of having a fever episode than those in the supplemented group [RR = 1.28 (1.09–2.94), p = 0.030]. The mean number of malaria episodes per child was also significantly higher in the placebo group. The RR of malaria episodes was 43% higher in the placebo group [RR = 1.43 (1.10–2.40), p = 0.025].

**Table 2:** Supplementation impact on malaria and general morbidity

	Placebo	Supplemented	RR (95% CI)	p-value
Number of fever episodes (mean ± SD)	2.04 ± 1.2	1.63 ± 1.2	-	0.038
Number of malaria episodes (mean ± SD)	1.5 ± 1.005	1 ± 0.93	-	0.029
^P*P^Fever episodes	153	119	1.28 (1.09–2.94)	0.030
^P*P^Malaria episodes	106	74	1.43 (1.10–2.40)	0.025
Free of pathologies	17% (9–25)	31% (16–46)	-	0.05
Cough episodes	51,3 (40–62.6)	45,9% (34.6–57.2)	-	0.510
Diarrhea episodes	52,7% (41.4–64)	28,3% (18.1–38.5)	-	0.002
Geometric mean density *P falciparum *(asexual forms/μl)	10672 (7589–15007)	7704 (5409–10971)	-	0.049

^P*P^Number of episodes

Mean ± SD or % (CI 95%)

Child-years was 37 in each group

**Figure 2:**
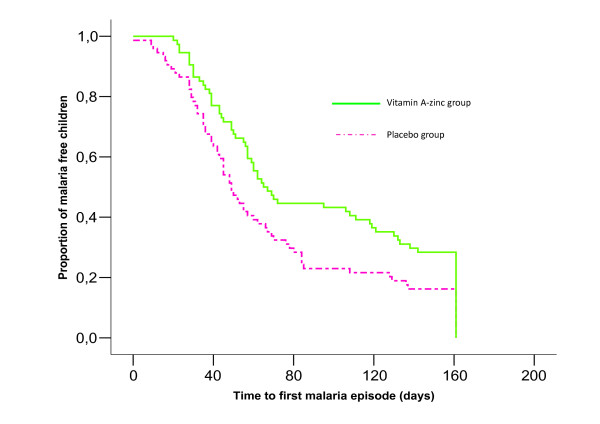
Kaplan-Meier survival analysis for the first clinical malaria episode for supplemented and placebo groups of children (Log Rank; p = 0.016). The continuous and green line represents the group of children who's received the double supplementation with VA and zinc. The dashed purple line represents the placebo group of children.

The Cox proportional hazards analysis (Figure 2) showed that the time to first episode of clinical malaria was significantly longer in the supplemented group than the placebo group (p = 0.016). Diarrhea episodes incidence were significantly higher in placebo group (52.7 vs. 28.3 p = 0.002). But no significant differences were observed for the incidence of cough between the two groups. Children in the supplemented group were more likely to be free from any episode of infection than those in placebo group (p = 0.05) (Table 2).

## Discussion

In this randomized trial we observed that combined supplementation with vitamin A and zinc reduced the burden of clinical attacks of malaria in young children by 30%. These results suggest that vitamin A and zinc supplementation might play an important role in the prevention of malaria. Vitamin A alone previously has been demonstrated to lead to a reduction of malaria episodes by 30% in Papua New Guinea [11]. Similarly, it was also observed that zinc supplementation alone reduced *P. falciparum*-attributable health attendance by 38% and by 69% for malaria episodes with high parasite density ≥ 100 000/μl [10]. When considering these previous studies [10,11], our results are suggestive of a potential synergistic effect of vitamin A and zinc on the reduction malaria episodes, even with low levels of parasitemia. However, because there were no groups of participants who received vitamin A or zinc alone in the present study, the study design precludes determining whether the reduction in malaria episodes was additive or multiplicative (i.e., synergistic). Mean parasite density observed during the follow up period tended to occur at higher densities in the placebo group. Prolongation of the time to first malaria episode manifestation was observed in one zinc supplementation study [10] but not in a vitamin A supplementation trial [11], so this lends support to a zinc-specific effect. Zinc appears to have an effect beyond an impact on malaria alone as many studies have shown that zinc reduces morbidity due to numerous infectious diseases [19-22]. Vitamin A seems to have a positive impact in terms of the number of malaria cases, independently from the *P. falciparum *parasitemia [9]. However, in previous studies in Ghana and in Burkina Faso, no effect was found on the number of falciparum malaria episodes or on mean parasite density with the supplementation of zinc alone or vitamin A alone [13,14].

One important limitation of our study was the imbalance at randomization between the two study arms. For example, the supplemented group had more children who were parasitemic at baseline, more who had a history of fever, and a greater proportion who were anemic. While the radical cure with SP should have resulted in the elimination of these differences between the two groups, it is possible that a greater intensity of past exposure to malaria in the intervention group might have resulted in a greater degree of acquired immunity which in turn could have led to a slower rate of re-infection. However, another possibility is that the study results are underestimating the true effect of the combined vitamin A and zinc supplements because the intervention group might have been subjected to a greater intensity of malaria transmission.

We observed that the number of fever episodes was significantly lower in children who received the supplements. In Papua New Guinea there was no effect of vitamin A alone on the number of fever episodes [11]. This confirms that the mechanism of action on malaria morbidity of vitamin A or zinc remains complex. It has been demonstrated that free retinol has a pharmacological effect against malaria parasites [23], but the very low concentrations of free retinol in the serum make its hypothetical effect inconclusive [24] given the lack of association between serum retinol concentration and malaria morbidity found in Papua New Guinea [11]. In another zinc supplementation study in Papua New Guinea [10], it was observed that plasma zinc levels alone were not predictive of susceptibility to malaria episodes, and despite the moderate improvement of plasma zinc levels in the supplemented group, malaria-associated morbidity was strongly reduced. These findings suggest that daily intake of zinc could modify resistance to *P. falciparum *episodes though pathways, perhaps at the cellular level, that are not fully reflected in plasma zinc levels [10].

It is known that vitamin A has immunopotentiating activities [6]; zinc also is essential for normal immune function [25]. Consequently, dual supplementation of these two micronutrients may lead to enhanced acquisition of immunity against falciparum malaria or to modified resistance to malaria episodes through a pathway at the cellular level [22]. These ideas are supported by the fact that the mean number of fever and malaria episodes was significantly lower in the supplemented group. Supplementation with vitamin A as well as zinc, or a combination of the two, is recognised to have a positive impact on anaemia [26-29], as observed in the current study. Improvements in anaemia might have resulted in reduced risk of fever and malaria rather than an immunododulating effect of the two micronutrients. Children in the supplemented group were more likely to be free from any pathological episode. Vitamin A supplementation provided simultaneously with expanded program on immunization (EPI) campaigns adopted by most of African in accordance with the recommendations of UNICEF represents a useful strategic approach to address the problem of vitamin A deficiency. However current strategies have not taken zinc supplementation into consideration with the notable exception of diarrhoea treatment programs. Ours findings suggest that dual supplementation with vitamin A and zinc is effective against malaria, and that a longer supplementation period might lead us to observe more important beneficial effects. Ultimately, the more affordable and sustainable solution would be the incorporation of vitamin A and zinc in food fortification for children.

## Conclusion

This study disclosed the key role of micronutrients in the reduction of children morbidity and mortality, liked to infectious diseases and malaria in particular. Our results suggest that combined supplementation with vitamin A and zinc may effectively reduce malaria-associated morbidity, and thus may play an important role in malaria control strategies in African children.

## Competing interests

The author(s) declare that they have no competing interests.

## Authors' contributions

ANZ coordinated the data collection entry, analysis and drafted the paper. NR, HS, IZ, and JR were the assistant of ANZ in the study, for data collection and laboratory analyses. NM from the International atomic energy agency (IAEA) was the technical advisor for the study and contributed to reviews of the paper. DHH provided technical input to the analysis and revisions of the manuscript. JBO, the director of the institute, designed the study with NM, coordinated the manuscript draft, and supervised the whole activities of the study. All authors approved the final manuscript.
